# The effectiveness of Problem Management Plus at 1-year follow-up for Syrian refugees in a high-income setting

**DOI:** 10.1017/S2045796024000519

**Published:** 2024-10-25

**Authors:** Anne M. de Graaff, Pim Cuijpers, Mariam Elsawy, Sam Hunaidy, Barbara Kieft, Noer Gorgis, Jos W. R. Twisk, Yenovk Zakarian, Theo K. Bouman, Miriam J. J. Lommen, Ceren Acarturk, Richard Bryant, David McDaid, Naser Morina, A-La Park, Peter Ventevogel, Marit Sijbrandij

**Affiliations:** 1 Department of Clinical, Neuro- and Developmental Psychology, WHO Collaborating Center for Research and Dissemination of Psychological Interventions, Amsterdam Public Health Research Institute, Vrije Universiteit Amsterdam, Amsterdam, The Netherlands; 2 Babeș-Bolyai University, International Institute for Psychotherapy, Cluj-Napoca, Romania; 3 ARQ National Psychotrauma Centre, ARQ Centrum ‘45, Diemen, The Netherlands; 4 i-Psy, Parnassia Groep, Almere, The Netherlands; 5 Department of Epidemiology and Data Science, VU University Medical Centre Amsterdam, Amsterdam, The Netherlands; 6 Clinical Psychology and Experimental Psychopathology, University of Groningen, Groningen, The Netherlands; 7 Department of Psychology, Koc University, Istanbul, Turkiye; 8 School of Psychology, University of New South Wales, Sydney, Australia; 9 Care Policy and Evaluation Centre, Department of Health Policy, London School of Economics and Political Science, London, UK; 10 Department of Consultation-Liaison Psychiatry and Psychosomatic Medicine, University Hospital Zurich, University of Zurich, Zurich, Switzerland; 11 Public Health Section, United Nations High Commissioner for Refugees, Geneva, Switzerland

**Keywords:** depression, posttraumatic stress disorder, randomised controlled trials, task sharing

## Abstract

**Aims:**

Problem Management Plus (PM+) has been effective in reducing mental health problems among refugees at three-month follow-up, but there is a lack of research on its long-term effectiveness. This study examined the effectiveness of PM+ in reducing symptoms of common mental disorders at 12-month follow-up among Syrian refugees in the Netherlands.

**Methods:**

This single-blind, parallel, controlled trial randomised 206 adult Syrians who screened positive for psychological distress and impaired functioning to either PM+ in addition to care as usual (PM+/CAU) or CAU alone. Assessments were at baseline, 1 week and 3 months after the intervention and 12 months after baseline. Outcomes were psychological distress (Hopkins Symptom Checklist [HSCL-25]), depression (HSCL-25 subscale), anxiety (HSCL-25 subscale), posttraumatic stress disorder symptoms (PCL-5), functional impairment (WHODAS 2.0) and self-identified problems (PSYCHLOPS).

**Results:**

In March 2019–December 2022, 103 participants were assigned to PM+/CAU and 103 to CAU of which 169 (82.0%) were retained at 12 months. Intention-to-treat analyses showed greater reductions in psychological distress at 12 months for PM+/CAU compared to CAU (adjusted mean difference −0.17, 95% CI −0.310 to −0.027; *p* = 0.01, Cohen’s *d* = 0.28). Relative to CAU, PM+/CAU participants also showed significant reductions on anxiety (−0.19, 95% CI −0.344 to −0.047; *p* = 0.01, *d* = 0.31) but not on any of the other outcomes.

**Conclusions:**

PM+ is effective in reducing psychological distress and symptoms of anxiety over a period up to 1 year. Additional support such as booster sessions or additional (trauma-focused) modules may be required to prolong and consolidate benefits gained through PM+ on other mental health and psychosocial outcomes.

## Introduction

The number of refugees worldwide has more than doubled over the past decade (UNHCR, 2022). An estimated 32% of refugees live with depression, and 31% with posttraumatic stress disorder (PTSD) (Patanè *et al.*, 2022). The prevalence of clinical depression and PTSD in displaced Syrians has been estimated to be 41% and 43%, respectively (Peconga and Høgh Thøgersen, 2020). The availability of (suitable) mental health services for refugees remains, however, low (Satinsky *et al.*, 2019). The majority of refugees are hosted by low- and middle-income countries where health systems are already under-resourced (Cratsley *et al.*, 2021), whereas health systems of higher income countries are typically limited by waitlists, and a lack of professionals speaking other languages and limited interpretation services (Satinsky *et al.*, 2019). As a result, many refugees in need of services are untreated (Fuhr *et al.*, 2020).

To address the mental health treatment gap (i.e., the proportion of individuals with a mental health condition that is not being treated for it) worldwide, the World Health Organization (WHO) developed a series of potentially scalable psychological interventions that can be implemented by non-specialists (WHO, 2017). Problem Management Plus (PM+) is a concise, transdiagnostic intervention aimed at alleviating symptoms of common mental disorders (CMDs) in individuals affected by adversity such as armed conflict (WHO, 2018). It consists of five sessions that cover skills related to managing stress, problem solving, behavioural activation and accessing social support (Dawson *et al.*, 2015). Research has demonstrated that PM+ delivered to individuals and groups in low-income settings has beneficial mental health effects 3 months after receipt of the intervention (2022a; Bryant *et al.*, 2017; Jordans *et al.*, 2021; Rahman *et al.*, 2016, 2019; Schäfer *et al.*, 2023). In high-income settings, non-specialist (or ‘task-shared’) interventions such as PM+ could be useful as a first step in a stepped-care model to scale up mental healthcare for refugees (Sijbrandij *et al.*, 2017). A recent study also demonstrated the effectiveness of individual PM+ for refugees over the same time period in a high-income setting (de Graaff *et al.*, 2023).

Although the evidence-base for potentially scalable interventions such as PM+ is growing, we lack evidence on whether such brief interventions (for refugees) can also have mental health benefits over the long term. A meta-analysis of trials examining the effects of psychosocial interventions for refugees and asylum seekers demonstrated intervention effects for depression, anxiety and PTSD directly after the intervention and at follow-up (any assessment 1 month after the intervention or longer). However, only 8 out of 26 studies included follow-up assessments longer than 3 months after the intervention (Turrini *et al.*, 2019). The first study that investigated long-term effects of group PM+ showed that initial treatment gains at 3 months were not sustained at 12-month follow-up among refugees within a closed refugee camp in Jordan (Bryant *et al.*, 2022b). Other research on PM+ thus far has been limited to the analysis of 3-month follow-up data (2022a; Bryant *et al.*, 2017; de Graaff *et al.*, 2023; Jordans *et al.*, 2021; Rahman *et al.*, 2016, 2019).

The randomised controlled trial (RCT) on individual PM+ for Syrian refugees in the Netherlands demonstrated that at 3-month follow-up, PM+ led to greater reductions in depression, anxiety, symptoms of PTSD and self-identified problems, but not functional impairment (de Graaff *et al.*, 2023). The current study reports on the 12-month follow-up (i.e., 12 months after baseline) of this trial with the aim to investigate whether PM+ reduces psychological distress (depression/anxiety combined), depression, anxiety, PTSD symptoms, functional impairment and self-identified problems at the long-term.

## Methods

### Design

A two-arm, single-blind RCT was conducted in the Netherlands at the Vrije Universiteit Amsterdam (VU) in collaboration with i-Psy mental healthcare. This study was undertaken within an international research consortium investigating scalable psychological interventions among Syrian refugees in Europe and the Middle East (Sijbrandij *et al.*, 2017). The trial was approved by the Research Ethics Review Committee at VU Medical Center (NL61361.029.17) and prospectively registered in the Netherlands Trial Registry (#7552). The Consolidated Standards of Reporting Trials (CONSORT) guideline can be found in Supplement A (Schulz *et al.*, 2010).

### Procedures

Adult (≥18 years) Arabic-speaking Syrian refugees were recruited through community centres, non-governmental organisations, reception centres, language schools and social media. By ‘Syrian refugees’, we refer to individuals from Syria who requested asylum after the start of the war in 2011 regardless of current resident status. Oral and written informed consent were obtained from all participants before screening (with a required 1 week ‘reflection time’). Inclusion criteria were psychological distress and functional impairment determined by a score ≥16 on the 10-item Kessler Psychological Distress Scale (K10) (Kessler *et al.*, 2002; Sulaiman-Hill and Thompson, 2010) and a score ≥17 on the 12-item WHO Disability Assessment Schedule (WHODAS 2.0) (WHO, 2010) (cf. Bryant *et al.*, 2017; Rahman *et al.*, 2016).

Exclusion criteria were acute medical conditions, imminent suicide risk (PM+ manual suicidality assessment), expressed acute needs/protection risks, indications of severe mental disorders (e.g., psychotic disorders), cognitive impairment (e.g., severe intellectual disability) as assessed by the PM+ manual observation checklist (World Health Organization, 2018) or current receipt of specialist mental healthcare. Participants meeting exclusion criteria were referred to the general practitioner/specialist services as required.

Participants were assessed at baseline, 1 week and 3 months after the intervention and 12 months after baseline (i.e., 10.5 months after the intervention). Assessments included Arabic-language questionnaires on demographics, clinical outcomes, daily functioning, stressful events and health service utilisation (de Graaff *et al.*, 2020a). Arabic-speaking assessors contacted participants for each assessment to share a personalized link to complete the online questionnaires using the questionnaire tool Survalyzer and to complete a phone-based interview on health service utilisation. Lower-literate participants were assisted by the audio-support function in Survalyzer or assistance from the assessor. Renumeration was 8.50 Euros for each follow-up assessment (totalling to 25.50 euros). Assessors were trained on questionnaire administration, general interview techniques, CMDs, psychological first aid and research ethics. Serious adverse events (SAEs) were recorded and monitored throughout the study.

Following baseline, participants were randomised on a 1:1 basis into PM+ in addition to care as usual (PM+/CAU) or CAU alone. An independent researcher not otherwise involved in the study generated a randomisation list with permuted block sizes of 4-6-8 in R (R Core Team, 2021), and a researcher not involved in the outcome assessments informed participants about the randomisation outcome using sealed opaque envelopes. Assessors completing outcome assessments were blinded to group allocation. Success of blinding was evaluated after each assessment (i.e., assessors indicated whether group allocation was revealed).

### Conditions

Detailed information on the intervention arms have been reported elsewhere (Dawson *et al.*, 2015; de Graaff *et al.*, 2020b, 2023). In brief, individual PM+ is a five-session intervention rooted in cognitive behavioural therapy and problem solving therapy delivered consecutively on a weekly basis with each session lasting 90 minutes. In the first four sessions, participants learn strategies for relaxation (i.e., slow breathing exercise), problem management, behavioural activation and accessing social support. Session five focuses on relapse prevention. Helpers were Arabic-speaking Syrian refugees with no formal training in mental health. Helpers completed an 8-day training in PM+ (including training on CMDs, basic counselling skills, delivery of the strategies and self-care) and received ongoing, weekly group supervision by trained PM+ supervisors (psychologists from i-Psy mental healthcare, VU and University of Groningen) during the trial. Sessions were delivered in the community (e.g. community centres, language schools), at VU, i-Psy mental healthcare or reception centres for asylum seekers. Following the implementation of COVID-19 restrictive measures (first partial lockdown in March 2020), participants were given the option for in-person or video call sessions. CAU comprised any (mental) health service available to refugees in the Netherlands (e.g., accessible through a general practitioner).

### Outcome measures

Psychological distress was measured by the Hopkins Symptom Checklist (HSCL-25). Items are scored on a 1–4 Likert scale with higher scores indicating worse symptomatology. For analysis, we used the mean of the items for the total scale and subscales. Probable depression and anxiety were defined by scores of 2.10 and 2.00 on the respective subscales (Mahfoud *et al.*, 2013). Functional impairment was measured by the 12-item WHODAS 2.0 (World Health Organization, 2010). Items are scored on a 1–5 scale (total range, 12–60). PTSD symptoms were assessed by the 20-item PTSD Checklist for DSM-5 (PCL-5) (Ibrahim *et al.*, 2018). Items are scored on a 0–4 scale (total range, 0–80), with a score of 33 or higher as indicator of probable PTSD (Bovin *et al.*, 2016). Self-identified problems were measured using the Psychological Outcomes Profiles (PSYCHLOPS) on a 0–5 scale (total range, 0–20) (Psychological Outcomes Profiles; PSYCHLOPS) (Ashworth *et al.*, 2004). All outcome assessments were administered at each time point.

### Other measures

Sociodemographic data included gender, age, living situation (i.e., living in a reception centre, independent housing in the community or other), education, marital status, work status, refugee status and time of displacement assessed through WHODAS 2.0. The number of potentially traumatic events was assessed with a dichotomously scored 27-item checklist (de Graaff *et al.*, 2020a). Post-migration stressors were assessed with the 17-item Post-Migration Living Difficulties (PMLD) checklist (Nickerson *et al.*, 2015). The total score was calculated by a count of all items with a score of at least 2 (moderately serious problem) (total score range, 0–17). Healthcare use was assessed using the Client Service Receipt Inventory (CSRI) (Chisholm *et al.*, 2000) adapted for this study.

### Statistical analysis

Power calculations for the trial were performed for the primary endpoint at 3-month follow-up. These were based on the results of the pilot RCT among Syrian refugees (de Graaff *et al.*, 2020b). No power calculations were performed for the 12-month follow-up, but the analysis was pre-planned and reported in the study protocol (de Graaff *et al.*, 2020a).

To measure baseline differences between the two conditions (i.e., PM +/CAU vs CAU) as well as between the retained and lost to 12-month follow-up sample, *t*-tests were conducted for continuous variables and Chi-squared tests for categorical variables. Additionally, a *t*-test was performed to test for any differences in the number of participants who reported having accessed mental healthcare services at 12-month follow-up. We also calculated the total number of contacts participants had with any mental health service. Mental health services were defined as services provided by a psychiatrist, psychologist, psychiatric nurse, self-help group, consultation centre, psychiatric crisis service, psychiatric outpatient service, mental health ward and long-stay psychiatric ward.

We performed linear mixed models (LMMs) in R on the intention-to-treat sample to estimate treatment effect at 12-month follow-up. The model included three dummy variables for time (i.e., post-assessment, 3-month follow-up and 12-month follow-up), three interaction terms for condition*time and a random intercept on subject level. In this model, the intercept reflects the baseline value for both conditions, because we did not include the main effect of condition to adjust for baseline differences between conditions (Twisk *et al.*, 2020). Regression coefficients of the interaction terms of condition with each follow-up time point are the effect estimates (i.e., mean difference between the two conditions). A separate model was estimated for the treatment effect at the whole follow-up period (i.e., the three follow-up time points on average).

We conducted LMMs for all outcomes, including psychological distress, depression, anxiety, functional impairment, symptoms of PTSD and self-identified problems. We conducted the same analyses with relevant covariates measured at baseline (i.e., gender, age, education, work status, number of potentially traumatic events, post-migration stressors and probable depression, anxiety and PTSD) to estimate covariate-adjusted models. These variables were also investigated as potential moderators (i.e., added in interaction with condition at 12-month follow-up) to the LMM of the HSCL-25 total score. Cohen’s *d* was calculated by the difference in least square means between conditions divided by the raw (i.e., as measured) pooled standard deviation (*SD*) at that assessment. A positive value for Cohen’s *d* indicates a beneficial effect for the PM+/CAU group in comparison to the CAU group.

Sensitivity analyses were performed including only participants retained at 12-month follow-up (completers) and including only participants of the PM+/CAU group who completed at least four sessions (per protocol) (de Graaff *et al.*, 2020b).

Across all analyses, two-tailed tests were reported where *p* < 0.05 indicated statistical significance.

## Results

### Participants

Participants were enrolled between March 2019 and December 2021 with the final 12-month assessments conducted in December 2022. There were 758 individuals who expressed interest in the project, of whom 236 signed informed consent and were screened. Of those, 206 met the inclusion criteria and completed baseline. Participants were randomised into PM+/CAU (*n* = 103) or CAU (*n* = 103). At inclusion, the sample was on average 36.5 years old (range, 18–69, *SD* = 11.7) and the majority (61.7%) were men (see Table 1).

**Table 1.** Baseline characteristics

	Full sample (*N* = 206)	PM+/CAU (*n* = 103)	CAU (*n* = 103)
Gender, *n* of men (%)	127 (61.7)	73 (70.9)	54 (52.4)
Age, *M* (*SD*) [range]	36.52 (11.72) [18−69]	36.35 (11.97) [18−69]	36.69 (11.52) [19−67]
Marital status, *n* (%)			
Never married	70 (34.0)	38 (36.9)	32 (31.1)
Currently married	99 (48.1)	51 (49.5)	48 (46.6)
Separated	4 (1.9)	2 (1.9)	2 (1.9)
Divorced	24 (11.7)	8 (7.8)	16 (15.5)
Widowed	5 (2.4)	2 (1.9)	3 (2.9)
Cohabiting	4 (1.9)	2 (1.9)	2 (1.9)
Work status			
Paid work	36 (17.5)	15 (14.6)	21 (20.4)
Non-paid work	30 (13.6)	17 (16.5)	13 (12.6)
Keeping house	7 (3.4)	5 (4.9)	2 (1.9)
Retired	2 (1.0)	1 (1.0)	1 (1.0)
Unemployed	40 (19.4)	14 (13.6)	26 (25.2)
Student (incl. language courses)	81 (39.3)	46 (44.7)	35 (34.0)
Other	10 (4.9)	5 (4.9)	5 (4.9)
Refugee status, *n* (%)			
Asylum procedure ongoing	16 (7.8)	10 (8.7)	6 (5.8)
Temporary resident permit	121 (58.7)	57 (55.3)	64 (62.1)
Permanent resident permit	29 (14.1)	14 (13.6)	15 (14.6)
Dutch citizenship	26 (12.6)	13 (12.6)	13 (12.6)
Other	2 (1.0)	2 (1.9)	0
Missing	12 (5.8)	7 (6.8)	5 (4.9)
Time elapsed (months) since arriving in the Netherlands,^a^ *M* (*SD*) [range]	44.07 (23.07) [1−113]	42.22 (23.57) [1−97]	45.94 (22.53) [2−113]
Educational level, *n* (%)			
No education	1 (0.5)	0	1 (1.0)
Basic education	29 (14.1)	10 (9.7)	19 (18.4)
Technical/vocational secondary	6 (2.9)	3 (2.9)	3 (2.9)
Technical diploma	13 (6.3)	7 (6.8)	6 (5.8)
Certificate of associate degree	18 (8.7)	11 (10.7)	7 (6.8)
General secondary education	37 (18.0)	21 (20.4)	16 (15.5)
Bachelor	82 (39.8)	41 (39.8)	41 (39.8)
Master	20 (9.7)	10 (9.7)	10 (8.7)
PhD	0	0	0
Depression and anxiety (HSCL-25 total), M (*SD*)	2.36 (0.62)	2.31 (0.64)	2.41 (0.61)
Depression (HSCL-25 subscale), *M* (*SD*)	2.47 (0.69)	2.41 (0.69)	2.52 (0.69)
Probable depression, *n* (%)^b^	142 (68.9%)	66 (64.1%)	76 (73.8%)
Anxiety (HSCL-25 subscale), *M* (*SD*)	2.20 (0.64)	2.16 (0.66)	2.24 (0.61)
Probable anxiety, *n* (%)^c^	129 (62.6)	55 (53.4)	74 (71.8)
PTSD symptoms (PCL-5), *M* (*SD*)	34.40 (16.93)	33.22 (17.84)	35.57 (15.96)
Probable PTSD, *n* (%)^d^	109 (52.9)	52 (50.5)	57 (55.3)
Functional impairment (WHODAS 2.0), *M* (*SD*)	29.46 (7.72)	29.09 (8.07)	29.84 (7.38)
Self-identified problems (PSYCHLOPS), *M* (*SD*)	15.55 (3.57)	15.38 (3.71)	15.72 (3.43)
Number of potentially traumatic events, *M* (SD) [range]	9.61 (5.09) [0−26]	9.91 (5.54) [0−26]	9.30 (4.61) [0−21]
Post-migration stressors (PMLD), *M* (SD) [range]	6.95 (3.55) [0−16]	6.74 (3.59) [0−16]	7.17 (3.51) [0−15]

PTSD = posttraumatic stress disorder.

a *n* = 200.

b Based on HSCL-25 depression subscale cut-off ≥2.10.

c Based on HSCL-25 anxiety subscale cut-off ≥2.00.

d Based on PCL-5 ≥33.

**Figure 1.**
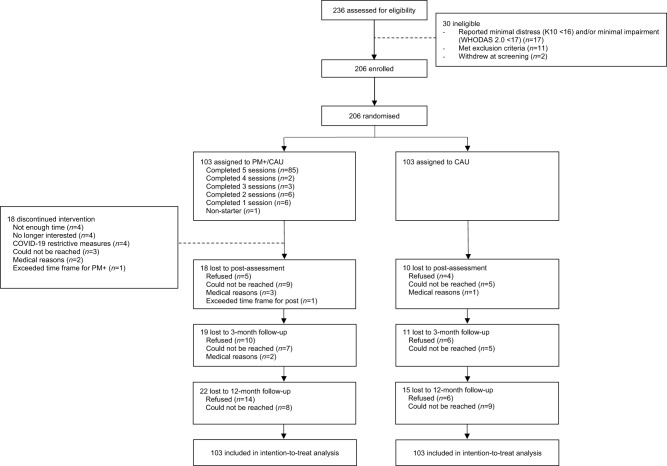
CONSORT flowchart.

We re-assessed 169 participants (82%) at 12-month follow-up, with 22 participants in PM+/CAU and 15 participants in CAU lost to follow-up (see flowchart in Figure 1). Of those, 20 (9.7%) refused to participate and 17 (8.3%) could not be reached. The loss to follow-up sample did not differ from the retained sample in terms of baseline characteristics (see Supplement B). At 12-month follow-up, blinding was successful in the assessment of 152 (89.9%) participants.

At 12-month follow-up, participants had on average spent 56 months (*SD* = 22.8, range, 11–109 months) in the Netherlands. The most frequently reported post-migration stressors were worries about family back home (65.7%), unable to return home in case of emergency (52.7%) and difficulties learning the Dutch language (50.9%) (see Table 2). The full sample reported an average of 5.1 (*SD* = 3.7) post-migration stressors at 12-month follow-up, which was significantly lower compared to the average number of 7.0 (*SD* = 3.6) post-migration stressors reported at baseline (estimate for main effect of time −1.63; 95% CI −2.208 to −1.042; *p* < 0.0001). There was no significant interaction between time and condition for the number of post-migration stressors reported at 12-month follow-up (mean difference −0.21; 95% CI −1.016 to 0.597; p = 0.61). The resident status of participants had also changed over the course of the trial. At baseline, 55/194 participants (28.3%) reported having a permanent resident permit or Dutch citizenship, 12 months later this was reported by 81/168 participants (48.2%).

**Table 2.** Post-migration stressors and healthcare use reported at 12-month follow-up

	Total (*N* = 169)	PM+/CAU (*n* = 81)	CAU (*n* = 88)
Post-migration stressors^a^			
Communication difficulties	53 (31.4%)	27 (33.3%)	26 (29.5%)
Discrimination	40 (23.7%)	18 (22.2%)	22 (25.0%)
Conflicts with own/other ethnic groups	15 (8.9%)	7 (8.6%)	8 (9.1%)
Separation from family	64 (37.9%)	31 (38.3%)	33 (37.5%)
Worries about family back home	111 (65.7%)	49 (60.5%)	62 (70.5%)
Unable to return home in emergency	89 (52.7%)	40 (49.4%)	49 (55.7%)
Difficulties with employment	64 (37.9%)	34 (42.0%)	30 (34.1%)
Difficulties in interviews immigration officials	16 (9.5%)	7 (8.6%)	9 (10.2%)
Conflict social workers/other authorities	24 (14.2%)	12 (14.8%)	12 (13.6%)
Not being recognised as a refugee	7 (4.1%)	4 (4.9%)	3 (3.4%)
Being fearful of being sent back	40 (23.7%)	17 (21.0%)	23 (26.1%)
Worries about not getting access to treatment	43 (25.4%)	17 (21.0%)	26 (29.5%)
Not enough money to pay for food, rent, clothes	49 (29.0%)	25 (30.9%)	24 (27.3%)
Difficulties obtaining financial assistance	30 (17.8%)	17 (21.0%)	13 (14.8%)
Loneliness, boredom, isolation	75 (44.4%)	35 (43.2%)	40 (45.5%)
Difficulties learning Dutch language	86 (50.9%)	41 (50.6%)	45 (51.1%)
Difficulties obtaining appropriate accommodation	44 (26.0%)	22 (27.2%)	22 (25.0%)
Healthcare use: Community services			
General nurse	17 (8.3%)	10 (12.3%)	7 (8.0%)
General practitioner	71 (34.5%)	38 (46.9%)	33 (37.9%)
Psychiatrist	11 (5.3%)	4 (4.9%)	7 (8.0%)
Psychologist	15 (7.3%)	9 (11.1%)	6 (6.9%)
Psychiatric nurse	4 (1.9%)	2 (2.5%)	2 (2.3%)
Social worker	9 (2.2%)	3 (3.7%)	6 (6.9%)
Physical therapist	21 (15.0%)	18 (22.2%)	13 (14.9%)
Home care	3 (1.5%)	2 (2.5%)	1 (1.1%)
Self-help group	2 (1.0%)	1 (1.2%)	1 (1.1%)
Consultation centre	0	0	0
Psychiatric crisis service	1 (0.5%)	1 (1.2%)	0
Other	6 (2.9%)	1 (1.2%)^b^	5 (5.7%)^c^
Healthcare use: Hospital care			
Mental health ward	0	0	0
Long-stay psychiatric ward	0	0	0
Other health ward	7 (3.4%)	4 (3.9%)	3 (2.9%)
Accident and emergency	2 (1.0%)	2 (1.9%)	0
Psychiatric outpatient service	0	0	0
Other (non-psychiatric) outpatient service	24 (11.7%)	12 (11.7%)	12 (11.7%)

Percentages represent valid percentages (i.e., based on number of retained participants).

a Three participants in the CAU group did not complete the questionnaire.

b Unclear *n* = 1.

c Dentist *n* = 3; public health centre *n* = 1; dietician *n* = 1.

Table 2 provides an overview of the different types of (mental) healthcare services that participants accessed in the three months preceding the 12-month follow-up. There was no statistically significant difference between the two groups in terms of the number of participants who accessed mental health services (*t* = −0.251, *p* = 0.8). In the PM+/CAU group, 11 participants (10.7%) had accessed mental health services with an average of 9.6 contacts (*SD* = 9.9; range, 1–30), while in the CAU group, 13 participants (12.6%) had accessed mental health services with an average of 5.9 contacts (*SD* = 4.8; range, 1–18).

Among participants in the PM+/CAU group, 85 (82.5%) completed all five sessions, two (1.9%) stopped after four sessions, three (2.9%) after three sessions, six (5.8%) after two sessions, six (5.8%) after one session and one (1.0%) did not complete any session. Due to COVID-19 restrictions, 64 (62.8%) participants completed the sessions in person, 25 (24.5%) online only (i.e., video calls) and 13 (12.7%) completed both in person and online (i.e., hybrid).

### Intervention effects

**Figure 2.**
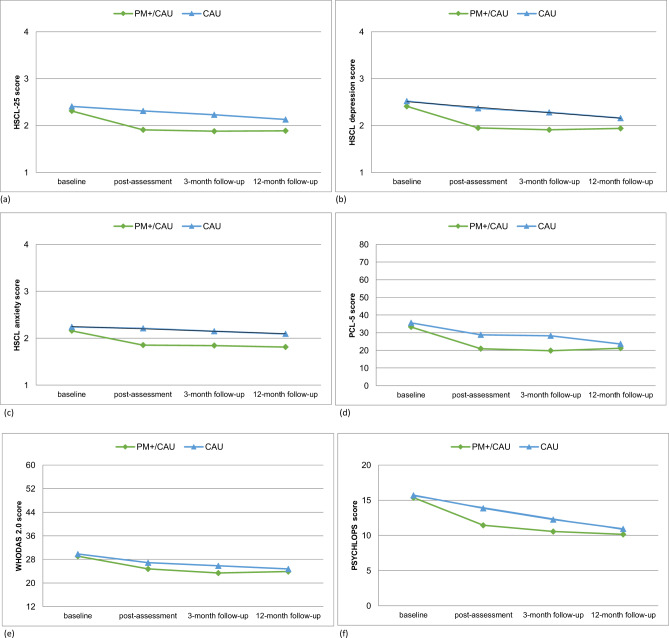
Group scores of primary and secondary outcomes across all time points. (a) HSCL-25 total, (b) HSCL depression, (c) HSCL anxiety, (d) PCL-5 PTSD symptoms, (e) WHODAS 2.0 functional impairment and (f) PSYCHLOPS self-identified problems.

The LMM analysis (see Table 3) showed that the intervention effect on the HSCL-25 total score (depression and anxiety combined) found at the primary endpoint at 3-month follow-up was sustained. At 12-month follow-up, the PM+/CAU group had a significantly larger reduction on the HSCL-25 total score compared with CAU (mean difference −0.17; 95% CI −0.310 to −0.027; *p* = 0.01; Cohen’s *d* = 0.28). The average scores for each condition on all outcomes are visualized in Figure 2.

**Table 3.** Summary statistics and results from the mixed-model analyses

		Descriptive statistics, *M* (*SD*)				Mixed-model analysis			Covariate-adjusted mixed-model analysis^c^		
Outcome	Time point	*n*	PM+/CAU (*n* = 103)	*n*	CAU (*n* = 103)	Difference in LS mean (95% CI)	*p*-value	Effect size^b^	Difference in LS mean (95% CI)	*p*-value	Effect size^b^
HSCL-25 Total score	Baseline	103	2.31 (0.64)	103	2.41 (0.61)						
	Overall effect^a^					−0.26 (−0.362, −0.148)	<0.0001	0.41	−0.21 (−0.301, −0.117)	<0.0001	0.33
	Post-assessment	85	1.91 (0.61)	93	2.31 (0.66)	−0.33 (−0.464, −0.190)	<0.0001	0.52	−0.28 (−0.406, −0.153)	<0.0001	0.44
	3-month follow-up	82	1.88 (0.61)	91	2.23 (0.63)	−0.26 (−0.398, −0.120)	0.0001	0.42	−0.21 (−0.341, −0.085)	0.001	0.34
	12-month follow-up	81	1.89 (0.54)	86	2.13 (0.68)	−0.17 (−0.310, −0.027)	0.01	0.28	−0.13 (−0.255, 0.005)	0.06	0.21
HSCL-25 Depression	Baseline	103	2.41 (0.69)	103	2.52 (0.69)						
	Overall effect^a^					−0.27 (−0.383, −0.148)	<0.0001	0.39	−0.22 (−0.326, −0.121)	<0.0001	0.31
	Post-assessment	85	1.95 (0.63)	93	2.37 (0.73)	−0.35 (−0.501, −0.198)	<0.0001	0.51	−0.31 (−0.445, −0.164)	<0.0001	0.45
	3-month follow-up	82	1.91 (0.63)	91	2.28 (0.69)	−0.28 (−0.434, −0.128)	<0.001	0.42	−0.24 (−0.383, −0.098)	0.001	0.36
	12-month follow-up	81	1.94 (0.62)	86	2.16 (0.73)	−0.15 (−0.310, 0.001)	0.05	0.23	−0.12 (−0.260, 0.028)	0.12	0.18
HSCL-25 Anxiety	Baseline	103	2.16 (0.66)	103	2.24 (0.61)						
	Overall effect^a^					−0.24 (−0.356, −0.133)	<0.0001	0.37	−0.19 (−0.284, −0.088)	0.0002	0.29
	Post-assessment	85	1.85 (0.65)	93	2.21 (0.64)	−0.30 (−0.444, −0.154)	<0.0001	0.45	−0.24 (−0.376, −0.107)	0.0005	0.37
	3-month follow-up	82	1.84 (0.64)	91	2.15 (0.64)	−0.23 (−0.377, −0.084)	0.002	0.36	−0.17 (−0.309, −0.036)	0.01	0.27
	12-month follow-up	81	1.81 (0.52)	86	2.09 (0.72)	−0.20 (−0.344, −0.047)	0.01	0.31	−0.14 (−0.277, 0.000)	0.05	0.22
PCL-5	Baseline	103	33.22 (17.84)	103	35.57 (15.96)						
	Overall effect^a^					−4.91 (−7.74, −2.084)	0.0007	0.37	−4.28 (−6.721, −1.852)	0.0007	0.25
	Post-assessment	85	20.89 (17.49)	92	28.80 (16.54)	−6.95 (−10.560, −3.335)	0.0001	0.41	−6.30 (−9.613, −2,967)	0.0002	0.37
	3-month follow-up	82	19.79 (16.59)	92	28.21 (16.38)	−6.56 (−10.200, −2.912)	0.0004	0.39	−5.81 (−9.157, −2.458)	0.0008	0.35
	12-month follow-up	81	21.22 (15.81)	85	23.59 (16.76)	−0.80 (−4.504, 2.913)	0.67	0.05	−0.37 (−3.811, 3.028)	0.83	0.02
WHODAS 2.0	Baseline	103	29.09 (8.07)	103	29.84 (7.38)						
	Overall effect^a^					−1.37 (−2.841, 0.101)	0.06	0.21	−0.66 (−1.991, 0.705)	0.34	0.08
	Post-assessment	85	24.76 (8.51)	93	26.90 (7.90)	−1.85 (−3.794, 0.086)	0.06	0.23	−1.09 (−2.916, 0.792)	0.25	0.13
	3-month follow-up	82	23.40 (8.25)	92	25.88 (7.38)	−1.74 (−3.711, 0.214)	0.08	0.21	−1.04 (−2.907, 0.837)	0.28	0.13
	12-month follow-up	81	23.89 (8.54)	87	24.77 (7.52)	−0.42 (−2.414, 1.565)	0.67	0.05	0.23 (−1.666, 2.140)	0.81	−0.03
PSYCHLOPS	Baseline	103	15.38 (3.71)	103	15.72 (3.43)						
	Overall effect^a^					−1.35 (−2.233, −0.472)	0.002	0.28	−1.07 (−1.898, −0.252)	0.01	0.22
	Post-assessment	85	11.44 (4.78)	92	13.86 (4.58)	−2.20 (−3.391, −1.008)	0.0003	0.47	−1.91 (−3.063, −0.754)	0.001	0.41
	3-month follow-up	84	10.56 (5.38)	91	12.25 (4.74)	−1.34 (−2.542, −0.144)	0.02	0.26	−1.04 (−2.208, −0.110)	0.08	0.21
	12-month follow-up	81	10.15 (4.55)	87	10.93 (4.60)	−0.39 (−1.606, 0.836)	0.53	0.09	−0.16 (−1.355, 1.012)	0.79	0.03

LS mean = Least Squares mean; PM+/CAU = Problem Management Plus in addition to care as usual; CAU = care as usual alone.

a This is the overall effect of condition on average over the two follow-up assessments

b Effect sizes were calculated using the difference in least square means between conditions divided by the raw pooled *SD* at that assessment. A positive value indicates a beneficial effect for the PM +/CAU group in comparison to the CAU group.

c Covariates included in these models are gender, age, education, marital status, work status and probable depression, anxiety and PTSD assessed at baseline, and trauma exposure and post-migration living difficulties assessed at baseline and 12-month follow-up.

We found a significantly larger reduction on HSCL anxiety in favour of the PM+/CAU group (−0.20; 95% CI −0.344 to −0.047; *p* = 0.01; *d* = 0.31), indicating that the intervention effect on anxiety at the primary endpoint was sustained over time. We did not find a significant difference between conditions on HSCL depression (−0.15; 95% CI −0.310 to 0.001; *p* = 0.05; *d* = 0.23). PM +/CAU also did not outperform CAU at 12-month follow-up on the PCL-5 (−0.80; 95% CI −4.504 to 2.913; *p* = 0.67; *d* = 0.05), WHODAS 2.0 (−0.42; 95% CI −2.414 to 1.565; *p* = 0.67; *d* = 0.05) and PSYCHLOPS (−0.39; 95% CI −1.606 to 0.836; *p* = 0.53; *d* = 0.09). This means that the short-term intervention effects on PTSD and self-identified problems at 3-month follow-up were no longer observed 12 months after baseline.

Covariate-adjusted models showed that the intervention effects at 12-month follow-up on HSCL-25 total and HSCL anxiety became less strong and were no longer significant (−0.13; 95% CI −0.255 to 0.005; *p* = 0.06; *d* = 0.21 and −0.14; 95% CI −0.277 to 0.000; *p* = 0.05; *d* = 0.22). The models for the other outcomes were consistent with the primary LMMs (Table 3).

Exploratory moderation analyses of the HSCL-25 total score showed a significant moderation of baseline depression (*p* = 0.007), with a stronger treatment effect for participants scoring above cut-off on the depression subscale at baseline (−0.25; 95% CI −0.395 to −0.096; *p* = 0.001; *d* = 0.53), than for participants scoring below cut-off on the depression subscale at baseline (0.01; 95% CI −0.446 to −0.071; *p* = 0.8; *d* = −0.05). Similarly, baseline anxiety (*p* < 0.0001) and PTSD (*p* = 0.0006) were significant moderators of the treatment effect on HSCL-25 total score, with a stronger effect for participants scoring above cut-off on anxiety (−0.30; 95% CI −0.456 to −0.140; *p* = 0.0002; *d* = 0.62) and PTSD (−0.33; 95% CI −0.495 to −0.169; *p* < 0.0001; *d* = 0.69), than participants scoring below cut-off on anxiety (0.08; 95% CI −0.079 to 0.248; *p* = 0.3; *d* = −0.22) and PTSD (0.02; 95% CI −0.176 to 0.143; *p* = 0.8; *d* = 0.04). We also found a negative effect modification with the number of potentially traumatic events at baseline (*p* = 0.004) and the number of post-migration stressors at baseline (*p* = 0.02), indicating that the effect of the intervention at 12-month follow-up became stronger with a higher number of potentially traumatic events and a higher number of post-migration stressors reported at baseline. Lastly, there was a significant moderation with gender (*p* = 0.03), with a stronger treatment effect for men (−0.23; 95% CI −0.385 to −0.081; *p* = 0.003; *d* = 0.38) than for women (−0.01; 95% CI −0.217 to 0.191; *p* = 0.9; *d* = 0.02). Participant characteristics such as age, educational level, marital status and work status did not moderate treatment effects (all *p* ≥ 0.05).

Sensitivity analyses of participants retained at 12-month follow-up and the per protocol sample showed that results were consistent with the primary LMMs (Supplements C and D). An exploratory subgroup analysis of the different PM+ delivery formats showed that participants who took in-person sessions (*n* = 64) had significantly lower HSCL-25 total scores relative to CAU at 12-month follow-up (−0.23; 95% CI −0.388 to −0.062; *p* = 0.007, *d* = 0.38), while participants who took video/hybrid PM+ sessions (*n* = 38) did not (−0.09; 95% CI −0.272 to 0.099; *p* = 0.36, *d* = 0.15) (Supplement E).

Four SAEs unlikely related to study procedures were reported (two in each group).

## Discussion

Against the backdrop of the rising number of forcibly displaced people worldwide, there is an urgent need to scale up psychological interventions for refugees and other displaced people. Although evidence supporting the short-term effectiveness of scalable interventions such as PM+ is growing (Bryant *et al.*, 2017; de Graaff *et al.*, 2023; Rahman *et al.*, 2016), little is known about their long-term effectiveness (Bryant *et al.*, 2022b). In the current study, we investigated whether PM+/CAU had beneficial effects on psychological distress, anxiety, depression, PTSD symptoms, functional impairment and self-identified problems at 1-year follow-up compared to CAU only in Syrian refugees living in the Netherlands.

A key finding was that PM+ had small but significant effects on the reduction of psychological distress and anxiety at 12-month follow-up. This is an important finding for scale-up given that PM+ was delivered by non-specialists in only five sessions. To date, there is limited evidence for the effectiveness of task-shared interventions (Karyotaki *et al.*, 2022; Singla *et al.*, 2017) and of psychological interventions examined among refugees and asylum seekers (Thompson *et al.*, 2018; Turrini *et al.*, 2021) beyond immediate post-assessment or 3-month follow-up. Although the gains on psychological distress were significant at 12 months, they were not as strong as what was found at 3-month follow-up (i.e., a reduction from *d* = 0. 41 at 3 months to *d* = 0.28 at 12 months) (de Graaff *et al.*, 2023), and became less strong and no longer significant when adjusted for covariates.

There was no statistically significant difference in depression scores at 12-month follow-up. That means that the initial treatment gains directly after the intervention and 3 months later (de Graaff *et al.*, 2023) were not sustained. However, it must be noted that this study was not powered for the 12-month follow-up and depression scores at 12 months were in favour of the PM+/CAU group. Moderation analysis showed that the reduction in psychological distress (i.e., depression and anxiety combined) was significant for men but not for women. It is unclear why women have no long-term benefits of PM+. Social factors may play a role or women’s vulnerability for a higher symptom burden over time (Musliner *et al.*, 2016). Additional support after PM+ such as booster sessions may help to sustain initial treatment effects, especially given the evidence for the long-term efficacy of problem-solving therapy on depression (Cuijpers *et al.*, 2021). PM+ may also be considered as a first step in a stepped-care model, whereby individuals who do not benefit from this brief intervention can be referred to specialist care (Sijbrandij *et al.*, 2017).

Contrary to our expectations, we did not observe a sustained intervention effect on symptoms of PTSD at 12 months. Participants who received PM+ reported a drop in PTSD symptoms immediately after the intervention and remained far below baseline level over time, while participants in CAU showed a gradual decrease in PTSD symptoms from baseline to 12-month follow-up. So, although we did not observe an intervention effect at 12 months, it appeared that participants receiving PM+ had a quicker decrease in PTSD symptoms compared with participants who did not receive PM+. However, although PTSD symptoms at 12-month follow-up remained far below baseline levels, the average symptom level was still considerable. Meta-analytic evidence examining psychosocial interventions among refugees and asylum seekers shows that trauma-focused psychological interventions are best supported for the reduction of PTSD symptoms (Nosè *et al.*, 2017; Turrini *et al.*, 2019). Complementing PM+ with a trauma-focused module may potentially enhance effectiveness for individuals with serious PTSD symptoms (Alozkan Sever *et al.*, 2021).

Similar to our findings at 1-week and 3-month follow-up, PM+ did not lead to reduced functional impairment at 12 months. ‘Functional impairment’ may be too generic as it also captures impairment due to medical conditions or social restrictions (e.g., during COVID-19 restrictive measures). The often challenging social context of refugees may have had a larger impact on their psychosocial functioning than the psychological intervention, especially over a 12-month period. A meta-analysis of psychosocial interventions for refugees and asylum seekers also demonstrated no treatment effects on functioning outcomes (Turrini *et al.*, 2019). We also found that the short-term benefits of PM+ in terms of reductions on self-identified problems (de Graaff *et al.*, 2023) were not retained. The effects of PM+ may be enhanced through integrated approaches, for example, if combined with social services that target social determinants of health including employment opportunities, housing and social support and better tap into the self-identified problems that often relate to practical issues.

Contrary to our expectation and to earlier findings where a higher number of post-migration stressors at baseline was associated with a smaller treatment effect at 3-month follow-up (Bryant *et al.*, 2022a; de Graaff *et al.*, 2023), higher numbers of potentially traumatic events and post-migration stressors at baseline were associated with larger treatment effects on psychological distress at 12 months. This may be explained by the association of potentially traumatic events and post-migration stressors with worse baseline symptom severity, which also modified the intervention effect at 12-month follow-up (i.e., participants scoring above clinical cut-offs had larger intervention effects).

Lastly, we found that participants who received in-person PM+ sessions had lower psychological distress compared to the control group, whereas participants who received video/hybrid PM+ sessions did not. These sub-group analyses on the delivery format of PM+ were in line with what was found for the short-term (de Graaff *et al.*, 2023). It must be noted that there was likely selection bias in the delivery format of PM+ because participants could choose (after an initial period in which no in-person sessions took place due to stricter COVID-19 measures) the format they preferred. Participants who took video/hybrid PM+ sessions had lower baseline scores compared to those who took in-person sessions, and it might be that participants with lower distress preferred the more easily accessible delivery format of video calls.

Strengths included high retention at 12-month follow-up (i.e., 82%) and the high representation of men in the sample which is rare in refugee studies. A limitation of this study was that mental healthcare utilisation was only reported for the preceding three months and did not cover the whole trial period. We thus do not know whether participants (in either group) accessed more specialist care for part of the study period. Another limitation is that the number of asylum seekers in this study was low (8% vs 85% of participants having a resident permit or citizenship), which prevented investigating whether resident status affected the effectiveness of PM+. Lastly, this study was not powered for a 12-month follow-up and no corrections for multiple testing were carried out, so conclusions about statistical significance should be interpreted with care.

An important implication of this study is that transdiagnostic and brief interventions like PM+ can be effectively delivered by non-specialists to refugees in high-income settings where PM+ could be considered a first step of stepped-care. Psychological therapies such as (trauma-focused) cognitive behavioural therapy focusing on a single disorder (e.g. PTSD) and delivered by specialists have better effect sizes (e.g., Weber *et al.*, 2021), but refugees in high-income countries may not access these services due to a lack of interpreters and long wait lists. We thus argue that, from a public health perspective, small intervention effects of non-specialist delivered interventions are crucial, especially as part of stepped-care and collaborative care models. In the past decade there has been a steep increase in the number of displaced people worldwide, including in European countries. There is an urgent need to scale up psychological interventions to meet the likely demand for mental health by refugees and other displaced populations. Booster sessions may be considered to enhance intervention effects over the long term, and implementation research is needed to evaluate the uptake of PM+ when integrated into healthcare systems.

## Conclusion

The benefits of PM+ delivered by non-specialist peer-providers to Syrian refugees on psychological distress and anxiety are sustained up to 1-year follow-up. To prolong benefits gained with PM+ on other mental health and psychosocial outcomes such as depression, PTSD symptoms and self-identified problems, stepping up to specialist support within a stepped-care model or the addition of targeted modules may be required. Considering the potential of PM+ for the long-term, scalable psychological interventions should be made available to underserved populations such as refugees.

## Supporting information

de Graaff et al. supplementary material 1 de Graaff et al. supplementary material

de Graaff et al. supplementary material 2 de Graaff et al. supplementary material

de Graaff et al. supplementary material 3 de Graaff et al. supplementary material

de Graaff et al. supplementary material 4 de Graaff et al. supplementary material

de Graaff et al. supplementary material 5 de Graaff et al. supplementary material

## Data Availability

Data are available on reasonable request. The Vrije Universiteit Amsterdam (VU) will keep a central data repository of all data collected in the STRENGTHS project. The data will be available on reasonable request to the STRENGTHS consortium. Data access might not be granted to third parties when this would interfere with relevant data protection and legislation in the countries participating in this project and any applicable European Union legislation regarding data protection. The PM+ training manual and intervention manual are available through the consortium. Interested researchers can contact Prof. Dr. Marit Sijbrandij at e.m.sijbrandij@vu.nl to initiate the process.

## Supplementary material

The supplementary material for this article can be found at https://doi.org/10.1017/S2045796024000519
