# Supplementary material for: The effectiveness of Problem Management Plus at 1-year follow-up for Syrian refugees in a high-income setting
Source: Epidemiol Psychiatr Sci. 2024 Oct 25;33:e50. doi: 10.1017/S2045796024000519 (PMC11588643; doi:10.1017/S2045796024000519)
Supplement: de Graaff et al. supplementary material 2 — de Graaff et al. supplementary material [file S2045796024000519sup002.docx]

**Supplement B**

| Table B1.  *Baseline Characteristics of Participants Retained and Lost at Follow-Up* | | | | |
| --- | --- | --- | --- | --- |
|  | Retained at 12-month follow-up (*n*=169) | Lost at 12-month follow-up (*n*=37) | t/χ^2^ | *p* |
| Gender, *n* of men (%) | 104 (62.0%) | 23 (62.2%) | 0.005 | 0.94 |
| Age, *M* (*SD*) [range] | 36.82 (11.33) | 35.14 (13.43) | 0.711 | 0.48 |
| Marital status, *n* (%) |  |  | 2.866 | 0.72 |
| Never married | 54 (32.0%) | 16 (43.2%) |  |  |
| Currently married | 83 (49.1%) | 16 (43.2%) |  |  |
| Separated | 3 (1.8%) | 1 (2.7%) |  |  |
| Divorced | 21 (12.4%) | 3 (8.1%) |  |  |
| Widowed | 4 (2.4%) | 1 (2.7%) |  |  |
| Cohabiting | 4 (2.4%) | 0 |  |  |
| Work status |  |  | 9.109 | 0.33 |
| Paid work | 33 (19.5%) | 3 (8.1%) |  |  |
| Non-paid work | 23 (13.6%) | 7 (18.9%) |  |  |
| Keeping house | 4 (2.4%) | 3 (8.1%) |  |  |
| Retired | 1 (0.6%) | 1 (2.7%) |  |  |
| Unemployed | 34 (20.1%) | 6 (16.2%) |  |  |
| Student | 65 (38.5%) | 16 (43.2%) |  |  |
| Other | 9 (5.3%) | 1 (2.7%) |  |  |
| Refugee status, *n* (%) |  |  | 6.635 | 0.24 |
| Asylum procedure ongoing | 10 (5.9%) | 6 (16.2%) |  |  |
| Temporary resident permit | 101 (59.8%) | 20 (54.1%) |  |  |
| Permanent resident permit | 24 (14.2%) | 5 (13.5%) |  |  |
| Dutch citizenship | 22 (13.0%) | 4 (10.8%) |  |  |
| Other | 1 (0.6%) | 1 (2.7%) |  |  |
| Missing | 11 (6.5%) | 1 (2.7%) |  |  |
| Time elapsed (months) at baseline since arriving  in the Netherlands,^a^ *M* (*SD*) [range] | 44.1 (22.6) [2-113] | 43.9 (25.5) [1-97] | 0.059 | 0.95 |
| Educational level, *n* (%) |  |  | 7.859 | 0.34 |
| No education | 0 | 1 (2.7%) |  |  |
| Basic education | 24 (14.2%) | 5 (13.5%) |  |  |
| Technical/vocational secondary | 5 (3.0%) | 1 (2.7%) |  |  |
| Technical diploma | 9 (5.3%) | 4 (10.8%) |  |  |
| Certificate of associate degree | 14 (8.3%) | 4 (10.8%) |  |  |
| General secondary education | 29 (17.2%) | 8 (21.6%) |  |  |
| Bachelor | 71 (42.0%) | 11 (29.7%) |  |  |
| Master | 17 (10.1%) | 3 (8.1%) |  |  |
| PhD | 0 | 0 |  |  |
| Depression and anxiety  (HSCL-25 total) | 2.35 (0.63) | 2.42 (0.60) | -0.658 | 0.51 |
| Depression (HSCL-25 subscale), *M* (*SD*) | 2.45 (0.69) | 2.55 (0.68) | -0.801 | 0.42 |
| Probable depression, *n* (%) ^b^ | 115 (68.0%) | 27 (73.0%) | 0.344 | 0.55 |
| Anxiety (HSCL-25 subscale), *M* (*SD*) | 2.20 (0.63) | 2.23 (0.66) | -0.314 | 0.75 |
| Probable anxiety, *n* (%) ^c^ | 106 (62.7%) | 23 (62.2%) | 0.004 | 0.94 |
| PTSD symptoms (PCL-5), *M* (*SD*) | 33.69 (16.42) | 37.62 (18.99) | -1.263 | 0.20 |
| Probable PTSD, *n* (%) ^d^ | 85 (50.3%) | 24 (64.9%) | 2.586 | 0.10 |
| Functional impairment (WHODAS 2.0), *M* (*SD*) | 29.20 (7.78) | 30.65 (7.44) | -1.005 | 0.31 |
| Self-identified problems (PSYCHLOPS), *M* (*SD*) | 15.62 (3.41) | 15.22 (4.24) | 0.625 | 0.53 |
| Number of traumatic events, *M* (SD) [range] | 9.87 (5.05) [0-18] | 8.41 (5.19) [0-26] | 1.591 | 0.11 |
| PMLD, *M* (SD) [range] | 6.89 (3.52) [2-15] | 7.22 (3.72) [0-16] | -0.500 | 0.61 |
| ^a^ *n*=203; PMLD = post-migration living difficulties; ^b^ based on HSCL-25 depression subscale cut-off ≥2.10; ^c^ based on HSCL-25 anxiety subscale cut-off ≥2.00; ^d^ based on PCL-5 ≥33; PTSD = posttraumatic stress disorder | | | | |
