# Supplementary material for: The effectiveness of Problem Management Plus at 1-year follow-up for Syrian refugees in a high-income setting
Source: Epidemiol Psychiatr Sci. 2024 Oct 25;33:e50. doi: 10.1017/S2045796024000519 (PMC11588643; doi:10.1017/S2045796024000519)
Supplement: de Graaff et al. supplementary material 3 — de Graaff et al. supplementary material [file S2045796024000519sup003.docx]

**Supplement C**

| Table C1. *Summary Statistics and Results from Mixed-Model Analysis of Primary and Secondary Outcomes for Retained Sample at 12-Month Follow-up* | | | | | | | | |
| --- | --- | --- | --- | --- | --- | --- | --- | --- |
|  |  |  | Descriptive statistics, *M* (*SD*) | | | Mixed-model analysis | | |
| Outcome | Time point | *n* | PM+/CAU (*n*=81) | *n* | CAU  (*n*=88) | Difference in LS mean (95% CI) | *p*-value | Effect size ^b^ |
| HSCL-25  Total | Baseline | 81 | 2.30 (0.65) | 88 | 2.39 (0.62) |  |  |  |
|  | Overall effect ^a^ |  |  |  |  | -0.27  (-0.381, -0.159) | <0.0001 | 0.43 |
|  | Post-assessment | 77 | 1.89 (0.59) | 84 | 2.30 (0.68) | -0.35  (-0.496, -0.207) | <0.0001 | 0.55 |
|  | 3-month follow-up | 77 | 1.87 (0.61) | 86 | 2.22 (0.65) | -0.28  (-0.424, -0.136) | 0.0001 | 0.44 |
|  | 12-month follow-up | 81 | 1.89 (0.54) | 86 | 2.13 (0.68) | -0.18  (-0.323, -0.038) | 0.01 | 0.29 |
| HSCL-25  Depression | Baseline | 81 | 2.41 (0.70) | 88 | 2.48 (0.69) |  |  |  |
|  | Overall effect ^a^ |  |  |  |  | -0.28  (-0.401, -0.157) | <0.0001 | 0.41 |
|  | Post-assessment | 77 | 1.94 (0.62) | 84 | 2.36 (0.76) | -0.37  (-0.531, -0.213) | <0.0001 | 0.53 |
|  | 3-month follow-up | 77 | 1.90 (0.63) | 86 | 2.26 (0.70) | -0.30  (-0.461, -0.144) | 0.0002 | 0.45 |
|  | 12-month follow-up | 81 | 1.95 (0.62) | 86 | 2.16 (0.73) | -0.17  (-0.322, -0.009) | 0.03 | 0.25 |
| HSCL-25  Anxiety | Baseline | 81 | 2.14 (0.65) | 88 | 2.25 (0.61) |  |  |  |
|  | Overall effect ^a^ |  |  |  |  | -0.26  (-0.378, -0.147) | <0.0001 | 0.41 |
|  | Post-assessment | 77 | 1.82 (0.62) | 84 | 2.22 (0.65) | -0.33  (-0.478, -0.175) | <0.0001 | 0.52 |
|  | 3-month follow-up | 77 | 1.82 (0.63) | 86 | 2.15 (0.66) | -0.25  (-0.404, -0.101) | 0.001 | 0.39 |
|  | 12-month follow-up | 81 | 1.81 (0.52) | 86 | 2.08 (0.72) | -0.21  (-0.358, -0.059) | 0.006 | 0.34 |
| PCL-5 | Baseline | 81 | 32.81 (17.31) | 88 | 34.50 (15.61) |  |  |  |
|  | Overall effect ^a^ |  |  |  |  | -5.50  (-8.387, -2.605) | 0.001 | 0.33 |
|  | Post-assessment | 77 | 20.12 (16.68) | 84 | 29.57 (16.75) | -8.15  (-11.865, -4.436) | <0.0001 | 0.49 |
|  | 3-month follow-up | 77 | 19.91 (16.75) | 87 | 28.20 (16.77) | -7.07  (-10.762, -3.382) | 0.0001 | 0.42 |
|  | 12-month follow-up | 81 | 21.22 (15.81) | 85 | 23.59 (16.76) | -1.27  (-4.940, 2.395) | 0.49 | 0.08 |
| WHODAS 2.0 | Baseline | 81 | 28.78 (8.15) | 88 | 29.59 (7.45) |  |  |  |
|  | Overall effect ^a^ |  |  |  |  | -1.42  (-2.960, 0.110) | 0.06 | 0.18 |
|  | Post-assessment | 77 | 24.69 (8.25) | 84 | 27.27 (8.05) | -2.28  (-4.331, -0.235) | 0.02 | 0.28 |
|  | 3-month follow-up | 77 | 23.69 (8.37) | 87 | 25.70 (7.44) | -1.55  (-3.582, 0.486) | 0.13 | 0.20 |
|  | 12-month follow-up | 81 | 23.89 (8.54) | 87 | 24.77 (7.52) | -0.48  (-2.494, 1.529) | 0.63 | 0.06 |
| PSYCHLOPS | Baseline | 81 | 15.31 (3.58) | 88 | 15.91 (3.24) |  |  |  |
|  | Overall effect ^a^ |  |  |  |  | -1.63  (-2.541, -0.723) | 0.0004 | 0.43 |
|  | Post-assessment | 77 | 11.19 (4.62) | 83 | 14.11 (4.34) | -2.65  (-3.882, -1.408) | <0.0001 | 0.59 |
|  | 3-month follow-up | 78 | 10.36 (5.44) | 86 | 12.35 (4.82) | -1.72  (-2.940, -0.491) | 0.006 | 0.33 |
|  | 12-month follow-up | 81 | 10.15 (4.55) | 87 | 10.93 (4.60) | -0.56  (-1.76, 0.652) | 0.36 | 0.12 |
| ^a^ This is the overall effect of condition on average over the three follow-up assessments; ^b^ Effect sizes were calculated using the difference in least square means between conditions divided by the raw pooled *SD* at that assessment. | | | | | | | | |
