# Supplementary material for: The effectiveness of Problem Management Plus at 1-year follow-up for Syrian refugees in a high-income setting
Source: Epidemiol Psychiatr Sci. 2024 Oct 25;33:e50. doi: 10.1017/S2045796024000519 (PMC11588643; doi:10.1017/S2045796024000519)
Supplement: de Graaff et al. supplementary material 4 — de Graaff et al. supplementary material [file S2045796024000519sup004.docx]

**Supplement D**

| Table D1. *Summary Statistics and Results from Mixed-Model Analysis of Primary and Secondary Outcomes for Per Protocol Sample* | | | | | | | | |
| --- | --- | --- | --- | --- | --- | --- | --- | --- |
|  |  |  | Descriptive statistics, *M* (*SD*) | | | Mixed-model analysis | | |
| Outcome | Time point | *n* | PM+/CAU (*n*=103) | *n* | CAU  (*n*=103) | Difference in LS mean (95% CI) | *p*-value | Effect size ^b^ |
| HSCL-25  Total | Baseline | 87 | 2.29 (0.67) | 103 | 2.41 (0.61) |  |  |  |
|  | Overall effect ^a^ |  |  |  |  | -0.26  (-0.368, -0.150) | <0.0001 | 0.42 |
|  | Post-assessment | 82 | 1.91 (0.61) | 93 | 2.31 (0.66) | -0.33  (-0.473, -0.194) | <0.0001 | 0.52 |
|  | 3-month follow-up | 79 | 1.88 (0.62) | 91 | 2.23 (0.63) | -0.26  (-0.404, -0.121) | 0.0002 | 0.42 |
|  | 12-month follow-up | 78 | 1.88 (0.55) | 86 | 2.13 (0.68) | -0.17  (-0.314, -0.028) | 0.01 | 0.28 |
| HSCL-25  Depression | Baseline | 87 | 2.40 (0.73) | 103 | 2.52 (0. 69) |  |  |  |
|  | Overall effect ^a^ |  |  |  |  | -0.27  (-0.386, -0.146) | <0.0001 | 0.40 |
|  | Post-assessment | 82 | 1.96 (0.63) | 93 | 2.37 (0.73) | -0.35  (-0.505, -0.197) | <0.0001 | 0.51 |
|  | 3-month follow-up | 79 | 1.91 (0.63) | 91 | 2.27 (0.69) | -0.28  (-0.439, -0.127) | <0.0001 | 0.42 |
|  | 12-month follow-up | 78 | 1.94 (0.62) | 86 | 2.16 (0.73) | -0.15  (-0.310, 0.005) | 0.06 | 0.22 |
| HSCL-25  Anxiety | Baseline | 87 | 2.13 (0.67) | 103 | 2.24 (0.61) |  |  |  |
|  | Overall effect ^a^ |  |  |  |  | -0.26  (-0.368, -0.141) | <0.0001 | 0.41 |
|  | Post-assessment | 82 | 1.85 (0.66) | 93 | 2.21 (0.64) | -0.31  (-0.459, -0.166) | <0.0001 | 0.48 |
|  | 3-month follow-up | 79 | 1.83 (0.64) | 91 | 2.15 (0.64) | -0.24  (-0.388, -0.090) | 0.001 | 0.37 |
|  | 12-month follow-up | 78 | 1.79 (0.52) | 86 | 2.09 (0.72) | -0.21  (-0.357, -0.055) | 0.007 | 0.34 |
| PCL-5 | Baseline | 87 | 32.90 (18.40) | 103 | 35.57 (15.96) |  |  |  |
|  | Overall effect ^a^ |  |  |  |  | -4.81  (-7.684, -1.934) | 0.001 | 0.29 |
|  | Post-assessment | 82 | 21.04 (17.56) | 92 | 28.80 (16.54) | -6.76  (-10.417, -3.105) | 0.0003 | 0.40 |
|  | 3-month follow-up | 79 | 19.76 (16.72) | 92 | 28.22 (16.38) | -6.66  (-10.352, -2.976) | 0.0004 | 0.40 |
|  | 12-month follow-up | 78 | 21.41 (16.04) | 85 | 23.59 (16.76) | -0.56  (-4.314, 3.192) | 0.77 | 0.03 |
| WHODAS 2.0 | Baseline | 87 | 29.11 (8.27) | 103 | 29.84 (7.38) |  |  |  |
|  | Overall effect ^a^ |  |  |  |  | -1.24  (-2.737, 0.261) | 0.10 | 0.15 |
|  | Post-assessment | 82 | 24.95 (8.60) | 93 | 26.90 (7.90) | -1.71  (-3.684, 0.259) | 0.09 | 0.21 |
|  | 3-month follow-up | 79 | 23.62 (8.30) | 92 | 25.88 (7.38) | -1.53  (-3.527, 0.462) | 0.13 | 0.19 |
|  | 12-month follow-up | 78 | 23.94 (8.67) | 87 | 24.77 (7.52) | -0.39  (-2.410, 1.634) | 0.71 | 0.05 |
| PSYCHLOPS | Baseline | 87 | 15.17 (3.92) | 103 | 15.72 (3.43) |  |  |  |
|  | Overall effect ^a^ |  |  |  |  | -1.27  (-2.174, -0.369) | 0.005 | 0.26 |
|  | Post-assessment | 82 | 11.55 (4.76) | 92 | 13.86 (4.58) | -2.10  (-3.312, -0.887) | 0.0007 | 0.45 |
|  | 3-month follow-up | 80 | 10.51 (5.43) | 91 | 12.25 (4.74) | -1.32  (-2.547, -0.099) | 0.03 | 0.26 |
|  | 12-month follow-up | 78 | 10.23 (4.53) | 87 | 10.93 (4.60) | -0.26  (-1.503, 0.983) | 0.68 | 0.06 |
| ^a^ This is the overall effect of condition on average over the three follow-up assessments; ^b^ Effect sizes were calculated using the difference in least square means between the PM+/CAU and CAU group divided by the raw pooled *SD* at that assessment. | | | | | | | | |
