# Supplementary material for: The effectiveness of Problem Management Plus at 1-year follow-up for Syrian refugees in a high-income setting
Source: Epidemiol Psychiatr Sci. 2024 Oct 25;33:e50. doi: 10.1017/S2045796024000519 (PMC11588643; doi:10.1017/S2045796024000519)
Supplement: de Graaff et al. supplementary material 5 — de Graaff et al. supplementary material [file S2045796024000519sup005.docx]

**Supplement E**

| Table E1.  *Summary Statistics and Results from Mixed-Model Analysis of Primary Outcome for Different PM+ Delivery Formats* | | | | | | | | | | | | | |
| --- | --- | --- | --- | --- | --- | --- | --- | --- | --- | --- | --- | --- | --- |
|  |  | Descriptive statistics, *M* (*SD*) | | | | | | Mixed-model analysis | | | | | |
|  |  | PM+/CAU | | | | CAU | | Difference in Least Squares mean (95% CI) | | | | | |
| Outcome | Time point | *N* | In-person sessions  (*n*=64) | *N* | Video/hybrid sessions  (*n*=38) | *N* | CAU  (*n*=103) | PM+ in-person sessions vs CAU | *p*-value | Effect size ^b^ | PM+ video/hybrid sessions vs CAU | *p*-value | Effect size ^b^ |
| HSCL-25 | Baseline | 64 | 2.41 (0.64) | 38 | 2.14 (0.60) | 103 | 2.41 (0.61) |  |  |  |  |  |  |
|  | Overall effect ^a^ |  |  |  |  |  |  | -0.32 (-0.448, -0.200) | <0.0001 | 0.50 | -0.15 (-0.296, -0.013) | 0.03 | 0.25 |
|  | Post-assessment | 50 | 1.91 (0.63) | 35 | 1.91 (0.59) | 93 | 2.31 (0.66) | -0.37 (-0.493, -0.241) | <0.0001 | 0.61 | -0.23 (-0.412, -0.050) | 0.01 | 0.36 |
|  | 3-months follow-up | 48 | 1.86 (0.62) | 34 | 1.90 (0.60) | 91 | 2.23 (0.63) | -0.35 (-0.507, -0.183) | <0.0001 | 0.55 | -0.14 (-0.318, -0.047) | 0.14 | 0.22 |
|  | 12-months follow-up | 48 | 1.89 (0.50) | 33 | 1.89 (0.61) | 86 | 2.13 (0.68) | -0.23 (-0.388, -0.062) | 0.007 | 0.38 | -0.09 (-0.272, 0.099) | 0.36 | 0.15 |
| ^a^ This is the overall effect of condition on average over the two follow-up assessments; ^b^ Effect sizes were calculated using the difference in least square means between the PM+/CAU and CAU group divided by the raw pooled *SD* at that visit. | | | | | | | | | | | | | |
